# The Mechanism of Metabolic Influences on the Endogenous GLP-1 by Oral Antidiabetic Medications in Type 2 Diabetes Mellitus

**DOI:** 10.1155/2020/4727390

**Published:** 2020-06-16

**Authors:** Thiquynhnga Nguyen, Min Gong, Song Wen, Xinlu Yuan, Chaoxun Wang, Jianlan Jin, Ligang Zhou

**Affiliations:** Department of Endocrinology, Shanghai Pudong Hospital, Fudan University, Shanghai 201399, China

## Abstract

Incretin-based therapy is now a prevalent treatment option for patients with type 2 diabetes mellitus (T2DM). It has been associated with considerably good results in the management of hyperglycemia with cardiac or nephron-benefits. For this reason, it is recommended for individuals with cardiovascular diseases in many clinical guidelines. As an incretin hormone, glucagon-like peptide-1 (GLP-1) possesses multiple metabolic benefits such as optimizing energy usage, maintaining body weight, *β* cell preservation, and suppressing neurodegeneration. However, recent studies indicate that oral antidiabetic medications interact with endogenous or exogenous GLP-1. Since these drugs are transported to distal intestine portions, there are concerns whether these oral drugs directly stimulate intestinal L cells which release GLP-1, or whether they do so via indirect inhibition of the activity of dipeptidyl peptidase-IV (DPP-IV). In this review, we discuss the metabolic relationships between oral antihyperglycemic drugs from the aspect of gut, microbiota, hormones, *β* cell function, central nervous system, and other cellular mechanisms.

## 1. Introduction

**Figure 1:**
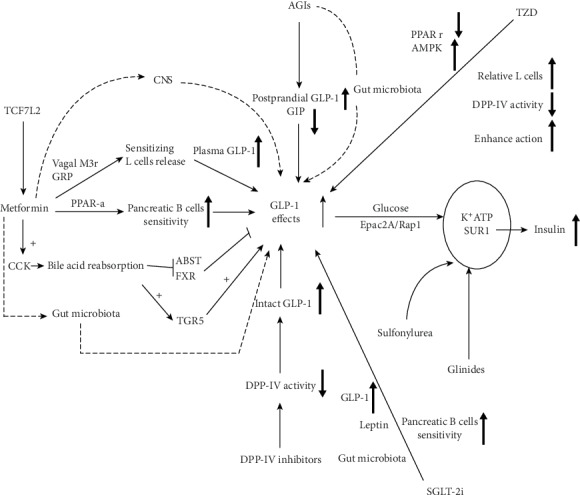
An overview of possible mechanisms of current major types of oral antidiabetic medications (OADs) on GLP-1 effect: including metformin, AGIs, TZD, SU, Glinides, SGLT-2i, and DPP-IV inhibitors. CNS: central nervous system; M3r: muscarinic receptor 3; GRP: gastrin-releasing peptide; PPAR-*α*: peroxisome proliferators-activated receptor; CCK: cholecystokinin; ABST: apical sodium-dependent bile acid transporter; FXR: farnesoid X receptor; TGR5: Takeda G protein-coupled receptor; AGIs: *α*-Glucosidase inhibitors; DPP-IV: dipeptidyl peptidase-IV; TZD: thiazolidinedione; AMPK: Adenosine 5′-monophosphate- (AMP-) activated protein kinase; Epac2A: the exchange protein directly activated by cAMP; Rap1: Ras-associated protein 1; K^+^ATP: ATP sensitive potassium channel; SUR1: sulfonylurea receptor-1; SGLT-2i: sodium-glucose cotransporter-2 inhibitor.

Type 2 diabetes mellitus (T2DM) is now the commonest type of diabetes mellitus in most economically developed nations. In recent years, the incidence of obesity and metabolic syndrome among younger populations has risen. T2DM is therefore emerging as a major public issue because it is not only difficult to control but also leads to multiple cardiovascular complications. According to the epidemic report by the International Diabetes Federation (IDF) published in 2017, there are an estimated 425 million people currently affected by diabetes. The report predicts that the number of people affected by diabetes will reach 629 million in the next 20 years [1]. In 2017, the prevalence rate of adult T2DM exceeded 10.9% in China, being highest among overweight and obese people [2]. To effectively control the high burden of diabetic nephropathy, coronary heart disease, and stroke in diabetic patients, new antidiabetic medications should be developed through preclinical and clinical research. In recent years, many antidiabetic drugs have been put into the market and several others are in the development pipeline most of which have shown good clinical benefits. Some of such drugs include incretin-based therapies and sodium-glucose cotranspotor-2 inhibitors (SGLT-2i). GLP-1 is an incretin that improves *β* cell function. This review describes the possible mechanisms and relationships between the currently used oral antidiabetic medications and GLP-1 (Figure 1). In doing so, we provide knowledge that can be used to guide the formulation of optimal treatment combinations for clinical management of T2DM.

## 2. Physiology of GLP-1

**Figure 2:**
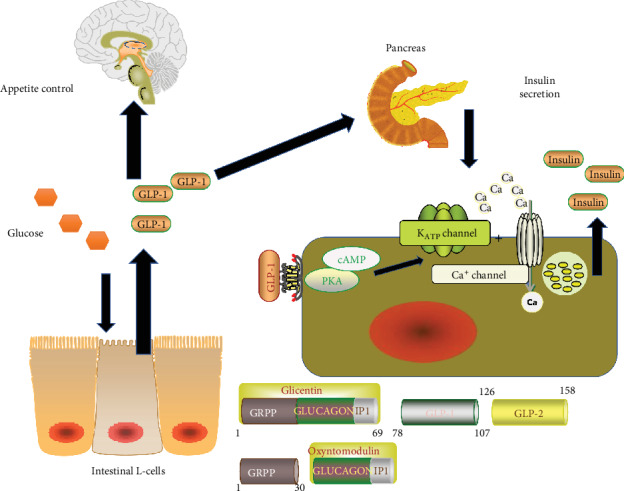
A brief review of the physiology of GLP-1. The intestinal GLP-1 can be secreted by the intestinal L cells under the stimulation of glucose. Then, the GLP-1 can bind to GLP-1 receptors of pancreatic *β*-cell. Its downstream action leads to the inhibition of ATP sensitive potassium channel, which results in the activation of calcium channel, and the accumulation of calcium in the cell promotes the secretion of insulin. On the other hand, the GLP-1 in the circulation can access the brain, which affects the appetite or energy control. cAMP: Cyclic Adenosine monophosphate; PKA: Protein kinase A; GRPP: Glicentin-related Pancreatic Polypeptide.

In the early 1900s, scientists discovered that the pancreas can produce endocrine substances, which lower blood glucose levels in response to the intestinal factors that triggered by meal stimulating [3]. These intestinal factors were subsequently named “incretin”. Later in the 1960s, scholars described the “incretin effect” by further confirming the existence of such endocrine factors. This effect states that oral glucose elicits a higher insulin level than intravenous administration in the context of the same level of blood glucose level [4]. It was later reported that this effect accounts for 50-70% of total insulin secretion. GLP-1 and glucose-dependent insulinotropic polypeptide (GIP) were later isolated from intestines, which led to the recognition that GLP-1 is responsible the majority activity of “incretin effect”. GLP-1 and glucagon are derived from a common preproglucagon gene but show tissue-specific patterns. After posttranslational processing by prohormone convertase 2 (PC2) or prohormone convertase 1/3 (PC1/3), respectively, preproglucagon gene generates several protein products such as Glicentin-related Pancreatic Polypeptide (GRPP), glucagon, major proglucagon fragment (MPGF), Intervening Peptide-1 (IP-1), few GLP-1, and GLP-2 in pancreatic *α* cells. In L-cells or PPG of the brainstem, the key products formed from proglucagon gene include glicentin, oxyntomodulin, GLP-1, and GLP-2 [5]. So far, only one receptor has been recognized to bind GLP-1. The classical GLP-1 receptor (GLP-1R) is a GPCR receptor widely expressed in several cells of the body. Its activation triggers diverse signaling cascades influencing various cellular functions (Figure 2). GLP-1R couples with adenylate cyclase thereby elevating intracellular cAMP levels leading to the activation of protein kinase A (PKA) [6] and cAMP-regulated guanine nucleotide exchange factor II (cAMP-GEFII, also known as Epac2) [7]. In this way, GLP-1 modulates the metabolic functions of target cells. The general effects of GLP-1 on metabolism include insulin release, inhibition of glucagon, *β* cell preservation, suppression of gastric emptying, anorexigenic, body weight reduction, bone formation, and organ protection (brain, heart, and kidney) [8].

Previous studies have demonstrated that oral drugs with GLP-1R agonists or DPP-IV inhibitors result in an unprecedented lowering of glucose compared to monotherapy and therefore improve metabolism. This has necessitated investigations into whether current oral medications have similar metabolic targets with GLP-1 signaling. In this review, we summarize the metabolic outcomes of oral antidiabetic medications with GLP-1-like effects.

## 3. Metformin and GLP-1

The absorption of metformin is a transporter-dependent process that mainly occurs in the small intestine [9]. Its uptake is through the apical (luminal-facing) surface of enterocytes via bidirectional transporters. Efflux across the basolateral surface of enterocytes is limited with passive diffusion through paracellular uptake resulting in the presence of metformin in the portal circulation. Several transporters have been identified including organic cation transporter (OCT) 1–3, plasma membrane monoamine transporter (PMAT), multidrug and toxin extrusion protein 1–2, serotonin transporter [6], and high-affinity choline transporter [6]. Genetic variation in OCT1 has been investigated by a number of groups, and these have generated useful insights into its pharmacokinetics, efficacy, and GI intolerance.

The mechanism of action of metformin with regards to its role in reducing hyperglycemia entails the lowering of hepatic glucose output and subsequently enhancing glucose uptake by skeletal muscles [10]. One of the critical genetic factors involved in this process is AMP-activated protein kinase (AMPK) [11]. Pathways involved in the lowering of glucose have been shown to be dependent on AMPK. For instance, its interaction with GLP-1 has been found to improve response to GLP-1 of pancreatic *β* cell via PPAR-*α* [12]. Meanwhile, metformin further enhances GLP-1 in the plasma. For instance, Mannucci et al. [13, 14] reported that metformin increases plasma concentrations of active GLP-1 in obese, nondiabetic, as well as in obese, diabetic subjects. In addition, the two trials (CAMERA and DIRECT) also reported that in nondiabetic individuals, metformin increases total GLP-1 in a sustained manner independent of changes in weight or glycaemia [15]. To date, it is still unclear whether this interaction is mediated by DPP-IV inhibition or GLP-1 secretion of metformin.

A recent study in which metformin (5-200 *μ*M) and AMPK activator aminoimidazole carboxamide ribonucleotide (100-1000 *Μ*m) were administered to murine human NCL-H716 and rat FRIC L cells revealed that neither of these agents stimulated GLP-1 secretion, indicating that metformin does not act on L cells to directly elicit secretion of GLP-1 [16]. Similarly, rodents orally or subcutaneously administered with metformin (300 mg/kg) and AMPK activator (250 mg/kg), respectively, showed a 2-8 fold increase in total plasma GLP-1 over a 2-hour period with no effect on the activity of DPP-IV. This indicates that the effect of metformin on *in vivo* GLP-1 plasma concentrations is regulated by increased peptide secretion. Moreover, pretreatment with a selective muscarinic 3 receptor antagonists also decreased GLP-1 levels. Bilateral subdiaphragmatic vagotomy revealed that only the basal GLP-1 levels were suppressed, and that a gastrin-releasing peptide acts as an antagonist blocking the metformin-induced GLP-1 secretion. These findings demonstrate that metformin stimulates the release of GLP-1 through a mechanism that involves muscarinic (M3) and GRP receptor-dependent pathways but is independent of both DPP-IV enzyme and direct effects on the intestinal L cell. Similarly, Thondam et al. [17] reported that chronic administration of metformin increased GLP-1 levels but with no effect on DPP-IV activity and Ghrelin levels. In addition, Vardarli and colleagues [18], while comparing the effect of sitagliptin and metformin on incretin, found that fasting significantly increased while an oral glucose challenge decreased total GLP-1 levels. However, intact GLP-1 increased in both fasting and postload by sitagliptin. Interestingly, only sitagliptin significantly augmented insulin secretion (in monotherapy and as an add-on to metformin), while the incretin effect was not changed numerically. This study suggests that metformin-induced GLP-1 release may not directly act on islet insulin secretion or via DPP-IV inhibition.

One of the links between metformin and GLP-1 release have been described using bile acids with a recent trial conducted to examine whether metformin suppressed bile acid reabsorption leads to GLP-1 secretion [19]. Here, it was reported that type 2 diabetic patients orally administered with metformin (1,500 mg) or placebo in combination with intravenous infusion of cholecystokinin (CCK) (0.4 mol/kg/min) or saline, showed an enhanced plasma GLP-1. It is understood that CCK induced excretion of bile acids that elicited release GLP-1 through action of a single dose of metformin. However, GLP-1-mediated induction of insulin or the release of glucagon was not observed [19]. This work further indicated that acute doses of metformin combined with bile acids improve glycemia and enhance GLP-1 release. Several *in vitro* studies have also shown that metformin can suppress the active reabsorption of bile acids in the terminal ileum by inhibiting apical sodium-dependent bile acid transporter (ASBT) [20, 21]. Subsequent processes such as the inhibition of nuclear farnesoid X receptor (FXR) and activation of the cell surface Takeda G protein-coupled receptor (TGR5) elicit GLP-1 secretion from L cells. Trabelsi et al. [22, 23] reported that the activation of FXR in L cells inhibits intracellular glycolytic pathways resulting in a reduction of GLP-1, whereas the activation of TGR5 induces GLP-1 secretion through increased ATP/ADP ratio and cyclic AMP levels in the L cells. The role played by TGR5 on GLP-1 secretion is best described by [24, 25], whose findings showed that plasma GLP-1 levels increased in patients with type 2 diabetes when bile acids were administered to the rectal or colon. In human trials, the specific ASBT inhibitors resulted in reduced levels of plasma glucose while GLP-1 concentrations increased in rodents [26, 27].

The rs7903146 T allele in transcription-factor-7-like-2 (TCF7L2), strongly associated with T2DM, has been described as an additional link between metformin response and GLP-1 levels [28]. Its factor binds to the promoter region of the proglucagon gene that encodes glucagon, GLP-1, and GLP-2. TCF7L2 exhibits an altered incretin signaling; potentially the mechanism of action with which it uses to increases T2D risk. As a result, it influences acute responses to both glipizide and metformin in people without diabetes. In the study, participants who carried the high-risk T allele at rs7903146 in the TCF7L2 gene showed higher fasting glucose levels during the baseline before glycemic perturbations. Similarly, high-risk T-allele carriers also had compensatory lower glucagon levels at baseline and higher GLP-1 levels displaying GLP-1 resistance.

Recently, other studies have reported that metformin may affect the gut-brain-liver axis [29] and even alter the composition of gut microbiota that in turn improve hyperglycemia [30, 31]. These open up new frontiers for further explorations into mechanisms of action of metformin on blood glucose metabolism.

## 4. Sulfonylurea (SUs), Glinides, and GLP-1

Insulin secretion is understood to be stimulated by SUs and GLP-1 of which calls for the need to unveil the common targets in the insulin secretion signaling. A recent direct target is the Epac2A/Rap1 signaling shared by GLP-1 and Sus [32]. Epac2A/Rap1 signaling is required for the first phase of glucose-induced insulin secretion. Epac2A, expressed in endocrine tissue, is both a PKA-independent pathway targets activated by GLP-1 and has binding sites for SUs. In this study, the researchers demonstrated that a combination of GLP-1 and Glibenclamide or Glimepiride augmented insulin secretion in Epac2A^+/+^mice and whereas a significant reduction was observed in Epac2A^−/−^mice. However, a combinatorial effect of GLP-1 and gliclazide was rather mild with the effect not altered by Epac2A ablation. A combination of an Epac-selective cAMP analog with glibenclamide or glimepiride favored the activation of Rap1 but not gliclazide. On the other hand, the ablation of Epac2A reduced the secretory response of insulin to the coadministration of the GLP-1 receptor agonist liraglutide and glimepiride in diet-induced obese mice. This study provides explanations on the effect of combining incretin-based drugs and SUs during clinical trials as they may easily induce episodes of hypoglycemia.

According to a majority of endocrinologists, the major triggering step to insulin secretion is the inactivation of K^+^ATP channels and the subsequent depolarization of *β*-cells [33, 34]. The K^+^ ATP channel is made up of four pore-forming Kir6.2 and four sulfonylurea receptor (SUR1) regulatory subunits. The Kir6.2 subunits act as glucose/ATP sensors by binding onto ATP in a Mg^2+^-dependent manner generate a conformational change that closes the channel. Conversely, the binding of Mg^2+^-ADP onto the SUR1 subunits opens the channel. At high glucose concentrations, the ATP/ADP ratio in the *β* cells increases, leading to channel closure and membrane depolarization, whereas a low glucose level elicits counteraction. SUs stimulates the secretion of insulin by the closing ATP-sensitive K^+^ channels and also directly bind to sulfonylurea receptor-1 (SUR1) to inhibit K(ATP) channels. On the other hand, GLP-1 binds onto the GLP-1 receptor stimulating *β*-cell cAMP formulation. Subsequent activation of PKA and Epac elicits downstream molecular activities related to insulin secretion including altered ion channel activity, elevation of intracellular calcium concentrations, and enhanced exocytosis of insulin-containing granules [35]. Effects of glucose and GLP-1 on insulin secretion may converge at the level of K(ATP) channels which is sensitive to present ATP levels, and PKA-mediated phosphorylation of S1448 in the SUR1 subunit. A result of this is that K(ATP) channel close via an ADP-dependent mechanism [5]. Such a phenomenon has been demonstrated *in vivo* where a targeted deletion of the SUR1 subunit resulted in the elevation of cAMP levels by GLP-1 but with no further stimulation of glucose-induced insulin secretion. The stimulation of insulin secretion by GLP-1 therefore relies on glucose metabolism by pancreatic *β* cells. At the same time, SUs may allow GLP-1R agonists to bypass glucose dependence by triggering *β*-cell depolarization even in the absence of glucose [36]. Therefore, SUs that bind SUR1 to modulate K(ATP) channels (independently of glucose) may influence GLP-1R agonists to uncouple the glucose-dependent manner by stimulating the downstream effects ordinarily associated with increased glucose.

Glinides do not activate Epac2A but nateglinide and mitiglinide have been shown to mobilize Ca^2+^ from the endoplasmic reticulum (ER) by binding onto the benzamido site of SUR1 on *β* cells. This is a separate site from that of the sulfonyl-binding but has a similar effect to sulfonylurea binding on the Kir6.2 channels. Despite this similarity, a relatively rapid onset and short duration of action restricts their use as prandial glucose-lowering agents [34, 37].

## 5. *α*-Glucosidase Inhibitors (AGIs) and GLP-1

AGIs selectively inhibit *α*-glucosidase enzymes in the brush border of enterocytes lining of the intestinal villi. This in turn prevents the cleaving of disaccharides or oligosaccharides into monosaccharides and postpones carbohydrate digestion and absorption leading to a reduction in blood glucose and lowering prandial insulin levels [37]. Previous studies have demonstrated that AGI treatment increases postprandial GLP-1 but reduces glucose-dependent insulinotropic polypeptide (GIP) secretions [38, 39]. Zhang et al. [40], when comparing treatment for T2D with acarbose and metformin, showed that both treatments notably increased the levels of GLP-1 but decreased those of glucagon after 24 weeks. Other studies have reported that chronic administration of acarbose significantly increased FGF21 levels whereas insulin-like growth factor-I (IGF-I) simultaneously decreased in serum, and this could be responsible for the observed extended lifespans in rodents [41]. Both hormones are mainly produced by the liver, and this phenomenon could be attributed to enhanced action of GLP-1 on hepatocytes which increases the expression of PPAR-*α* and Sirt1 to promote FGF21 production. Moreover, SCFAs mainly derived from undigested carbohydrates or insoluble fibers fermented by gut microbiota play an important role in AGIs treatment in patients with diabetes. Acarbose has previously been shown to increase the levels of serum butyrate (an SCFA) in individuals with impaired glucose tolerance [42]. An increase in SCFAs promotes GLP-1 production [43]. Voglibose has been found to possess antiobesity abilities which induce changes in dysbiosis in diet-induced obese mice. These compounds therefore increase bile acid metabolites and confer benefits on systemic outcomes such as cardiovascular end-points [44]. In addition, the administration of miglitol increases butyric acid levels in the intestines and suppresses colon inflammation [45]. The underlying mechanisms of AGIs action in improving metabolism have been comprehensively investigated and reported [46]. These include the effects of incretins, activation of the neuroendocrine response to leptin, and induction of expression of the genes responsible for the enhancement of energy metabolism.

## 6. Thiazolidinedione and GLP-1

Thiazolidinedione (TZDs) are agonists of peroxisome proliferator-activated receptor (PPAR); a nuclear receptor that regulates the transcription of genes involved in lipid and glucose metabolism [47]. Although predominantly expressed in adipose tissues, PPAR is present in other insulin-sensitive tissues including the liver, muscle, and pancreatic islet cells [48, 49]. In adipose tissues, the stimulation of PPAR increases adipocyte differentiation resulting in an increased number of small, insulin-sensitive adipocytes which improves glucotoxicity. The development of these insulin-sensitive cells in turn enhances glucose uptake, improving glycemic control. TZDs also promote FA uptake and storage in adipose tissue thus reducing levels of circulating FFAs. Functionally, this has the potential to alleviate lipotoxicity. TZDs protect *β* cell function and prevent apoptosis by improving insulin sensitivity (indirectly) and acting on PPAR*γ* in *β* cell of pancreatic islets (directly). Despite limited knowledge on the influence of TZDs on GLP-1, there have been attempts to describe the existence of metabolic relationships as well as the need to further explore abilities to preserve *β* cell function, affect lipid metabolism for application in the prevention of prediabetes.

For instance, a previous study in Zucker diabetic fatty rats reported that treatment of rats with metformin and pioglitazone significantly decreased DPP-IV activity in serum as well as glycosylated hemoglobin. Regression analysis further indicated that DPP-IV activity in serum positively correlated with glycosylated hemoglobin and negatively with GLP-1, respectively. However, *in vitro* studies showed that metformin, pioglitazone, and glyburide did not influence serum DPP-IV activity indicating these medications are not competitive DPP-IV inhibitors [50]. In addition, the study also found that DPP-IV activity increased with ageing in T2DM subjects, and that kidney and liver RNA levels were unchanged. Since DPP-IV is secreted in some tissues, a potential explanation for this is that decreased DPP-IV activity could be secondary to improved glycemic control which could regulate the release of DPP-IV from T-cells, endothelial, pancreatic islet *α*, or other types of cells. These could determine the extent to which these cells contribute to the pool of soluble DPP-IV activity.

Another research group seeking to determine whether insulin-sensitizing drugs improve secretion of GLP-1 studied catch-up growth rats that display insulin resistance and impaired incretin effect after pioglitazone treatment. They found that rats fed on high-fat diets showed improved insulin resistance, high levels of circulating GLP-1, and increased relative number of intestinal L cells [51]. However, the effect of TZDs was not comparable to metformin treatments known as an incretin release enhancer. A separate study examined whether the TZD rosiglitazone had direct action on pancreatic *β* cells in acute or long-term rosiglitazone in clonal pancreatic BRIN-BD11 *β* cells maintained in standard, glucotoxic, and lipotoxic cultures. The results showed that rosiglitazone (6.25 *μ*M) enhanced acute insulinotropic action of GLP-1, and displayed direct beneficial effects on *β* cell viability and function during gluco- or lipotoxicity setting [52]. Although the interaction of GLP-1 and TZDs have not been adequately investigated or elucidated so far, it is possible that this effect could be attributable to PPAR-*γ* agonist. This is because this compound can lead to the activation of adenylate cyclase or late events in G protein-coupled pathways, or the effect to the modulation of AMPK activity by TZDs as previously demonstrated. This was partially demonstrated by the action of SCFAs in protecting against high-fat diet-induced metabolic abnormalities regulated by PPAR*γ* repression. It is understood that this repression subsequently increased the expression of mitochondrial uncoupling protein 2 and AMP/ATP ratio which is responsible for activating AMPK, increasing oxidative metabolism in the liver and adipose tissues, and elevating levels of GLP-1 [53]. Moreover, the metabolic relationship was also demonstrated by the inverse influence of GLP-1 signaling on PPAR*γ* during lipid metabolism. Decara et al. [54] showed that chronic administration of Liraglutide regulator changed lipid metabolism in a diet and tissue-dependent manner in rats, resulting in its decrease in the liver and muscles of high fat diet-induced obesity rats but an increase in lean rats. Moreover, it has been shown that both GLP-1 and TZDs can be utilized as treatment options in nonacholic fatty liver disease (NAFLD) [55]. To compare the effect of pancreatic *β* cells protection, Kimura et al. [56] found that both GLP-1 agonists, Liraglutide and pioglitazone, preserved the function and mass of *β* cells, although this action was more profound during the early compared to advance stages in T2DM in db/db mice. In conclusion, TZDs treatment has metabolic impact upon GLP-1 action and shares some similarities despite their distinct action targets.

## 7. Sodium-Glucose Cotransporter-2 Inhibitors (SGLT-2i) and GLP-1

Sodium-glucose cotransporter-2 inhibitors (SGLT-2i) are major proteins responsible for the glucose reabsorption in the kidney. Although mainly expressed in the proximal tubule of the kidney, they also localize in other organs or tissues of the body. A recent preliminary study using SGLT-2 antibody immunohistochemistry detected their expression in multiple areas in the central nervous system, including the hypothalamus, periaqueductal grey, and nucleus of the solitary tract. The administration of SGLT-2i in mice could have an impact on nuclei activities in CNS related to autonomic neural regulation (unpublished data). However, the mechanism of its associated effects has not been elucidated. And this can be attributed to limited studies on the drug. Some studies have shown that SGLT-2i could function by inhibiting the absorption of glucose in the renal tubule, thus resulting in the excretion of large amounts of carbohydrates into the urine [57]. The mechanism presents advantages and limitations. The excretion of glucose accompanied by sodium and fluids induces negative energy metabolic status that could result in weight loss, cardiac protection, and renal protection. However, chronic glucose deficit and excess ketone in the blood might lead to excess food consumption and euglycemic diabetic ketoacidosis (euDKA). Nonetheless, this drug is beneficial to the metabolism of patients with T2DM because of its insulin-independent action that releases excessive energy status or insulin resistance. The drug is also associated with a low incidence of adverse effects. Multiple clinical trials have demonstrated the efficacy and safety of a combination of DPP-IV inhibitors and SGLT-2i in the treatment of T2DM.

A few studies have shown that SGLT-2i alone can elevate the circulation of GLP-1 in patients with T2DM. Takebayashi et al. reported that canagliflozin treatment alone significantly increased the area under the curve (AUC)[0-120 min] of active plasma GLP-1 after three days compared with that at baseline and the addition of teneligliptin (DPP-IV inhibitor) resulted in a further increase [58]. In this study, canagliflozin treatment raised the active GLP-1 plasma level in the early phase (30 min), probably by inhibition of SGLT-1 by low selective SGLT-1/SGLT-2 inhibitors that could have led to the increased levels of GLP-1 secreted by lower intestinal L cells. Also, other neuroendocrine factors could have been at play. This was consistent with another study in which the utilization of Empagliflozin (high selective for SGLT-2) also raised the plasma GLP-1 level in the early phase [59]. And SGLT-2i was found to improve the incretin sensitivity of pancreatic *β* cells in patients with T2DM. Chang et al. showed that in a 3-hour hyperglycemic clamp study involving incretin infusion before and after 8-week treatment with dapagliflozin, the C-peptide response to GLP-1 significantly increased and that to GIP/GLP-1 also increased (but not significantly) whereas both the insulin responses to GLP-1 and GIP/GLP-1 increased significantly [60].

Given that both SGLT-2 and GLP-1 are associated with weight control and food intake, Hiroshi et al. investigated the effect of SGLT-2 inhibitors by evaluating the effect of 16 weeks of ipragliflozin treatment on body weight and hormones related to appetite regulation in patients with BMI ≥ 22 kg/m^2^. The treatment led to a significant reduction in fasting serum leptin levels after two weeks, which remained the same for up to 16 weeks, whereas the plasma active ghrelin level showed no significant change [61]. The reduction of the leptin level could indicate the resolution of leptin resistance accompanied with bodyweight reduction because overweight or obesity is associated with leptin resistance. Numerous studies have demonstrated that GLP-1 interacts with leptin to influence food intake and body weight. The administration of GLP-1 agonists for a short period decreased circulating and free plasma leptin levels, which led to an increase in the soluble leptin receptors thus bodyweight reduction [62, 63]. Therefore, SGLT-2i and GLP-1 could partly share common neuroendocrinal mediators that influence body weight.

Concerning the effects on CNS, both SGLT-2i and GLP-1 have shown neuroprotection potential. Canagliflozin administration was shown to improve glucose and lipid metabolism by indirectly attenuating obesity-induced inflammation in the nodose ganglion, hypothalamus, and skeletal muscle of mice [64], thus reducing the symptoms of metabolic syndrome, which could have been accomplished by the GLP-1. Although the current study did not investigate the effect of SGLT-2i in the CNS, a few studies have shown that it could have antiseizure properties because of its low molecular weight and liposolubility [65], and antidegenerative brain diseases [66]. SGLT-2i could also gain entry into the brain by permeating the blood-brain barrier or via another neuroendocrinal pathway to influence autonomic activities such as cardiovascular regulation (unpublished data).

The gut has been used widely for the study of metabolic syndrome [67]. The occurrence of metabolic syndrome disorders has been associated with increased expression of SGLT-2 [68]. However, a study indicated that SGLT-2i could improve the gut microenvironment. In the study, diabetic animals treated with dapagliflozin showed a decrease in the gut microbiota composition, such as Firmicutes/Bacteroidetes ratio and Oscillospira, and an increase in *Akkermansia muciniphila* [69], which suggested that the gut microbiota composition could be influenced by SGLT-2i and GLP-1interaction.

## 8. DPP-IV Inhibitors and GLP-1

Glucagon-like peptide-1 (GLP-1) has a short life span in plasma (less than 2 minutes) because of degradation by DPP-IV and other enzymes. Therefore, incretin-based therapy in clinics uses DPP-IV inhibitors or modified GLP-1 analog/GLP-1 receptor agonists as an antihyperglycemic option for patients with T2DM [5]. Dipeptidyl peptides-4 (DPP-4) inhibition allows GLP-1 to remain longer in plasma and exerts its insulin release stimulatory activities. The DPP-IV inhibitors include sitagliptin, saxagliptin, alogliptin, vildagliptin, and linagliptin. It has been reported that DPP-IV inhibition leads to a 5 to 10 pmol/L increase in meal-induced plasma concentrations of intact GLP-1. These types of medications are well tolerated, and only a few adverse effects have been observed in clinical use. Studies have shown that these drugs also confer cardiovascular benefits.

Contrary to the GLP-1 analogs, DPP-IV inhibitors have a neutral effect on body weight and therefore do not cause weight loss. Also, they do not cause hypoglycemia, particularly when combined with GLP-1 analogs. Dipeptidyl peptides-4 is also known as the T-cell activation antigen CD26 and one of the enzymes responsible for GLP-1 breakdown. It is also involved in thymic maturation and migration patterns [70]. The inhibition of DPP-IV was found to alter the expression of the immune response-related genes in the thymus, especially those related to central immunological tolerance. The inhibition of DPP-IV could also contribute to the prevention of T1DM in NOD mice. Several studies have shown that DPP-4 inhibition in patients with T2DM has the potential to treat pulmonary hypertension by exerting anti-inflammatory activity [71]. Other studies have also revealed that DPP-4 inhibition has renoprotective effects independent of blood pressure and glucose-lowering effects [72]. Dipeptidyl peptides-4 has also been implicated in cancer development because of its tendency to promote tumor cell adhesion, migration, and metastasis. Furthermore, autoimmune diseases pathophysiology such as multiple sclerosis, tuberculoid leprosy, Graves' disease, systemic lupus erythematosus (SLE), rheumatoid arthritis, and the human immunodeficiency virus/acquired immunodeficiency syndrome (HIV/AIDS) are associated with CD26/DPP-IV activity [73]. Recently, reports have emerged that associate DPP-IV treatment with the onset of Bullous pemphigoid [74]. Thus, other than glycemic homeostasis, DPP- IV inhibitors play several roles in immunity. Currently, there is an increasing need for a more precise incretin regulation/better blood glucose control while minimizing adverse effects or complications.

## 9. The Side Effects and Expected Side Effects on Combination of OADs

At present, there are many drugs available for diabetes treatment, but most of the mechanisms of drug action cannot be separated from the role of insulin. Therefore, the hypoglycemic effect of conventional hypoglycemic drugs may be weakened or even some adverse reactions may occur under the condition of severe insulin deficiency or function defect. Traditional hypoglycemic drugs include metformin, sulfonylureas, glinides, *α*-glucosidase inhibitors, and thiazolidinediones. Among them, metformin may not be able to effectively control blood glucose because some people cannot tolerate gastrointestinal reaction, liver, and kidney function decline and cannot reach the standard dose in the case of lactate acidosis; sulfonylurea as an insulin secretagogues, although they have significant hypoglycemic effect, can cause the insecurity of hypoglycemia and can also cause weight gain. Insulin secretagogues may also increase the load of islet cells and accelerate islet apoptosis; therefore, they cannot be applied to patients producing diabetes-related antibodies, cases such as latent autoimmune diabetes in adults (LADA). In general, the combination of conventional OADs with an insulin secretagogue should be avoided in some populations in the case of hypoglycemia. AGIs can also cause adverse reactions of the digestive system, and hypoglycemic effects are limited. TZDs can cause sodium and water retention, thus, may increase risks of heart failure; SGLT-2i also induces potential genitourinary tract infections, or euglycemia ketonic acidosis, and may increase the risks of amputations. Finally, in type 1 diabetes and late-stage 2 diabetes, these drugs will be more difficult and lack of blood glucose control. There is also a lack of evidence-based support for cardiovascular safety and protection of conventional OADs.

## 10. Conclusion

The global prevalence of type 2 diabetes mellitus (T2DM) is rising steadily. And because of its close association with obesity, the rise in the incidence of the condition reflects the changing lifestyle of modern society. The disease is also associated with the onset of other disorders, especially those related to the cardiovascular system. However, numerous studies show that the condition can be managed at an early stage and its complications can also be reduced at an advanced stage. The management strategies of the disease include lifestyle changes such as diet and exercise, or the use of antidiabetic medications, which are regarded as noninvasive and safe. Currently, several drugs for managing T2DM are available, and each has its unique metabolic targets and intra- or extracellular mechanisms. The algorithms for the application of these drugs in clinics have been established in many guidelines proposed by diabetes associations of many countries, and the dosage administered is based on the individual glycemic and health status. Currently, the common goal of developing new antidiabetic medications is to minimize cardiovascular complications and confer more metabolic benefits beyond glycemic control. The current incretin-based therapy affects the ominous octet or more defects in the pathophysiology of T2DM. Many preclinical studies and clinical trials have demonstrated the efficacies and safety of this therapy or its combination treatments. Notably, studies have shown that drug combinations are more effective in reducing hyperglycemia, which may be attributed to the enhancement of the level of action of GLP-1.

In summary, the following areas were discussed in the present review. Firstly, this review concluded that metformin is a secretagogue or release sensitizer rather than a potent DPP-IV inhibitor. The AMPK, bile acid, genes related to T2DM and GLP-1, microbiota, and the gut-brain-liver axis influence the production, release, and *β* cell response of GLP-1. Secondly, the signaling and targets of GLP-1 and Sulfonylurea on insulin release were compared and based on the distinct action on the common K^+^ATP channels; multiple studies questioned the rationale or safety of the two-insulin stimulating drug combination. Thirdly, the results on the role of *α*-glucosidase inhibitor were compiled, the mechanisms of which majorly involves intestine, as an enhancer of the metabolic action of GLP-1. Next, the limited study results on Thiazolidinedione (TZDs) and its relation with GLP-1 were reviewed. The PPAR signaling and GLP-1 are mutually influenced and have similar effects on insulin release, *β* cell protection, cardiovascular benefits, and treatment of NAFLD, etc. Moreover, the mechanism of SGLT-2i as a novel insulin-independent medication was also discussed in the present review. The unique and versatile functions of this drug and its promotion of GLP-1 release or action were summarized. Also, the effects of the drug were compared with the action of GLP-1, including energy control and bodyweight reduction, CNS activities regulation, gut microenvironment. Finally, the impact of DPP-IV inhibition on GLP-1 and its essential role in immunity was discussed. Collectively, the new mechanisms of oral antidiabetic medication on the effect of GLP-1 are listed to reveal the recent progress and perspectives on the treatment, which can provide an understanding of new mechanisms and treatment options (Table 1). The findings of these studies provide the rationale for the development of new drugs or formulations, which can ensure better glucose metabolism.

## Figures and Tables

**Table 1:** Summarization on the relationships between oral antidiabetic medications and GLP-1.

OADs	Mechanisms on interactions with the GLP-1 effects	Related target molecules
Metformin	Majorly promote GLP-1 release and inhibits DPP-IV activity; Improves response to GLP-1 of pancreatic *β* cell Interact with gut-brain-liver axis; Affect microbiota	AMPK, PPAR-*α*; Bile acids absorption repression; TCF7L2
Sulfonylurea	Enhance insulin secretion and may induce hypoglycemia while combination	Epac2A/Rap1 of K^+^ATP channels
Glinides	Enhance insulin secretion, but short duration of action	Benzamido site on SUR1 of K^+^ATP channels,
*α*-Glucosidase inhibitor	Majorly promote GLP-1 release Endocrine modulation Affect microbiota	FGF21, IGF-I, hepatic PPAR-*α* and Sirt1
TZDs	Promote GLP-1 release and inhibits DPP-IV activity Endocrine modulation Improves response to GLP-1 of pancreatic *β* cell	PPAR-*γ*, AMPK
SGLT-2i	Promote GLP-1 release, endocrine modulation, affect microbiota	SGLT-2
DPP-IV i	Inhibit GLP-1 degradation	DPP-IV

OADs: oral antidiabetic drugs; TZDs: Thiazolidinedione; PPAR-*α*: peroxisome proliferators-activated receptor; DPP-IV: dipeptidyl peptidase-IV; AMPK: Adenosine 5′-monophosphate- (AMP-) activated protein kinase; Epac2A: the exchange protein directly activated by cAMP; Rap1: Ras-associated protein 1; K^+^ATP: ATP sensitive potassium channel; SUR1: sulfonylurea receptor-1; SGLT-2i: sodium-glucose cotransporter-2 inhibitor.
